# A behavioral task for investigating action discovery, selection and switching: comparison between types of reinforcer

**DOI:** 10.3389/fnbeh.2014.00398

**Published:** 2014-11-18

**Authors:** Simon D. Fisher, Jason P. Gray, Melony J. Black, Jennifer R. Davies, Jeffery G. Bednark, Peter Redgrave, Elizabeth A. Franz, Wickliffe C. Abraham, John N. J. Reynolds

**Affiliations:** ^1^Department of Anatomy, Brain Health Research Centre, University of OtagoDunedin, New Zealand; ^2^Department of Psychology, Brain Health Research Centre, University of OtagoDunedin, New Zealand; ^3^Department of Psychology, University of SheffieldSheffield, UK

**Keywords:** joystick, action, discovery, selection, learning, switching, basal ganglia

## Abstract

Action discovery and selection are critical cognitive processes that are understudied at the cellular and systems neuroscience levels. Presented here is a new rodent joystick task suitable to test these processes due to the range of action possibilities that can be learnt while performing the task. Rats learned to manipulate a joystick while progressing through task milestones that required increasing degrees of movement accuracy. In a switching phase designed to measure action discovery, rats were repeatedly required to discover new target positions to meet changing task demands. Behavior was compared using both food and electrical brain stimulation reward (BSR) of the substantia nigra as reinforcement. Rats reinforced with food and those with BSR performed similarly overall, although BSR-treated rats exhibited greater vigor in responding. In the switching phase, rats learnt new actions to adapt to changing task demands, reflecting action discovery processes. Because subjects are required to learn different goal-directed actions, this task could be employed in further investigations of the cellular mechanisms of action discovery and selection. Additionally, this task could be used to assess the behavioral flexibility impairments seen in conditions such as Parkinson's disease and obsessive-compulsive disorder. The versatility of the task will enable cross-species investigations of these impairments.

## Introduction

The survival of all animals—from nematodes to humans—largely depends on their ability to act appropriately in their environment. That is, to perform actions that lead to advantageous outcomes, and avoid those that do not. In this sense an action can be considered a movement, or sequence of movements, that causes a predicted outcome. The cognitive processes of action selection support this ability by providing a solution for a fundamental question: given the current internal and environmental states, what action is most likely to promote survival?

To select the most appropriate action, the agent must first acquire knowledge of the causal relationships between specific dimensions of behavioral output (“where,” “what,” “when,” and “how”) and particular outcomes. Through trial and error the system converges on the specific parameters of the different dimensions that are required to produce a particular outcome. This process, critical to action selection, can be termed action discovery or agency determination. During this process the system dynamically weights competing action possibilities according to the relative values of the outcomes they produce. Action-outcome connections are strengthened for those actions that lead to desirable outcomes, and weakened for those that produce punishment or lack of behavioral significance, which is the underlying basis of Thorndike's Law of Effect (Thorndike, 1911). Thus, actions associated with desirable outcomes are more likely to be selected again in the future under similar internal and environmental states.

The cellular mechanisms of action discovery and subsequent action selection are poorly understood at present. For instance, as the central nervous system deals with a constant stream of competing sensory inputs and motor outputs, an action discovery process must be able to determine which component of a recently performed action actually caused a particular unpredicted event to occur. Reinforcement signals associated with the caused event must also “work backwards” to modify the weighting of the putative action components, so that the critical causal components can be identified and an action-outcome association can be formed. These issues have been well characterized as the credit assignment problem (Minsky, 1961; Barto et al., 1983; Izhikevich, 2006). There is considerable evidence that the modification of synaptic circuits involving the basal ganglia plays an important role in this assignment problem (Redgrave et al., 2011). In particular, short-latency sensory inputs to ventral midbrain dopamine neurons together with corresponding inputs to the basal ganglia via subcortical loops are likely to be critically involved (Fisher and Reynolds, 2014).

On a more conceptual level, a related framework proposes that basal ganglia-thalamo-cortical circuits are integral to the allocation of attention so that goal-directed behaviors become possible (Franz, 2012). Support for the basal ganglia in this role has been demonstrated in rodents (Mink, 1996) and humans (Franz, 2006), and a wealth of findings support the involvement of these processes in procedural learning and switching of behavioral and cognitive sets (Hayes et al., 1998; Shook et al., 2005; Disbrow et al., 2013). The cognitive domains of task switching and reversal learning form part of the higher-level concept of behavioral flexibility, and are therefore related to action discovery and selection. These tasks have been studied extensively in animal models and humans. In rats, the prelimbic and orbitofrontal areas of cortex, and the dorsal striatum contribute to the flexible control of behavior (Ragozzino, 2007).

Toward the aim of elucidating cellular mechanisms of these complex processes, animal models employing behavioral tasks allowing for more complex responses are required. The current standard lever pressing or nose poking tasks, once acquired, require a new subject or a new task for further investigation to proceed. However, to investigate the processes of action discovery and action selection at behavioral and cellular levels, it would be advantageous to use repeated measures experimental designs, where multiple actions can be learnt and selections can be made between them. We present here a new joystick task that is fit for this purpose.

The use of joysticks to examine behavior has a long history in psychological and medical research. Their use with human participants (e.g., Leonard, 1953), non-human primates (e.g., Hopkins et al., 1992), and more recently rodents (e.g., Washburn et al., 2004), demonstrates the cross-species utility of joysticks. However, they have rarely been used to study action acquisition and selection. Combining the many degrees of freedom allowed by a joystick with well-designed experimental protocols creates an effective means of investigating action discovery and selection processes for several key reasons. First, a joystick has an advantage over more traditional lever or nose poke inputs because the parameters of the action required to elicit the reward can easily be changed. Thus, the target zone to which the joystick must be manipulated to trigger reward delivery can be altered while still maintaining the base knowledge of how to interact with the manipulandum. With each target change, a new action has to be discovered, and the subject must select, or switch, between these actions to achieve the goal. Therefore, a joystick allows for continuous new learning and switching, which are critical to the effective study of action discovery and selection. Second, the task difficulty can be modified dynamically within or between sessions simply by changing the size of the target area, thereby adding a further dimension of continuous learning. Moreover, joysticks can be used to acquire complex multi-action sequences, further adding to their versatility. A recently published joystick task designed for action discovery focuses on such longer searching actions, with a joystick that can be manipulated across a large space without automatically centering (Stafford et al., 2012).

The study presented here characterizes a self-centering (returns to center position on release) joystick task designed for rats. Different types of reinforcement were used: standard food pellets and electrical brain stimulation reward (BSR). BSR is of interest because the precise temporal control over reinforcement delivery and dose afforded by BSR can be exploited to study cellular mechanisms of action discovery and selection. The reinforcing properties of BSR are thought to be mediated by its effects on reward-related pathways, such as those of the dopamine neurons in the substantia nigra *pars compacta* (SNc) (Phillips and Fibiger, 1978; Fibiger et al., 1987). Results of early experiments with BSR suggested that there were behavioral differences in tasks reinforced with BSR in comparison to those with natural rewards such as food (Olds, 1958; Howarth and Deutsch, 1962). However, when task demands were more tightly controlled, for example by ensuring identical contingencies (such as timing and movements required) existed between response and reward delivery (Gibson et al., 1965), there were no longer differences in acquisition, response rate and extinction, at least in simple lever-pressing tasks.

We hypothesized firstly that rats will be able to learn to manipulate a joystick into multiple positions to initiate reinforcer delivery, and to adapt their movements to progress through target areas of increasing degrees of difficulty. Secondly, that rats will be able to discover new target positions and switch between these to meet changing task demands. Thirdly, that when the experimental contingencies are matched, the performance of rats reinforced with BSR will be similar to those reinforced with food. This study provides a proof of concept of the utility and versatility of this joystick task for investigating action discovery, selection and switching processes. As such it will be useful in further investigations of underlying cellular mechanisms, and how they are modified in abnormal states, including Parkinson's disease and obsessive-compulsive disorder.

## Materials and methods

### Groups

All procedures involving animals were approved by the University of Otago Animal Ethics Committee. Male Long-Evans rats, weighing between 260 and 350 g (7 to 10 weeks old) at the start of experimentation, completed the joystick task. Rats were maintained on a reverse light cycle, and experiments were performed during their night period. For one group (*n* = 9), rats received grain-based, dustless food pellets (Bio-Serv) as rewards for a correct target hit, and were food deprived for 12 h prior to experiments.

In a separate group of rats (*n* = 9), BSR was used in place of food reward. These animals (250–280 g, 7–9 weeks old) were first surgically implanted with a stimulating electrode. They were anesthetized with ketamine (75 mg/kg i.p.) and domitor (0.5 mg/kg i.p.), and administered prophylactic antibiotic (Amphoprim, 0.2 ml s.c.). A stainless steel twisted-pair stimulating electrode (MS303/2-B/SPC, Plastics One) was implanted into the left SNc (anteroposterior 4.6–5.0 mm, mediolateral 1.8–2.0 mm relative to bregma; dorsoventral 7.7 mm from brain surface) and secured with dental cement (Vertex Self-Curing). Five days were allowed for post-operative recovery before experimentation began.

BSR was delivered by a constant current stimulator (PHM-152, Med Associates Inc.) and comprised of a biphasic pulse, 500 μs in total width, applied at 100 Hz for 500 ms. A 500 μs pulse width was selected to maximize the activation of dopamine fibers (Grace and Bunney, 1983; Yeomans, 1989).

### Joystick hardware and software

**Figure 1:**
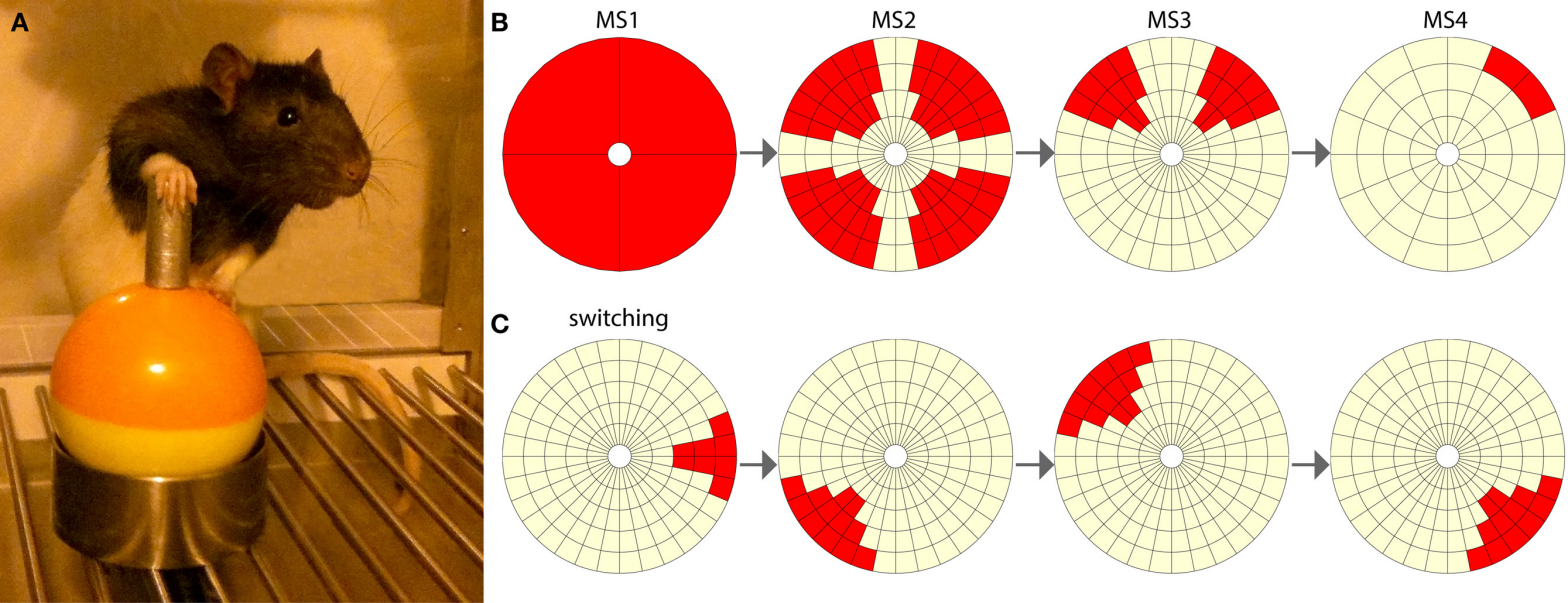
**The joystick task. (A)** Rat using the joystick in a typical position, with one paw driving the manipulandum and the other paw balancing on the inferior ball of the joystick. **(B)** Milestones 1 to 4 of increasing difficulty. The red segments represent targets, and the light-yellow segments are neutral. If the rat moves the joystick into a target area, a hit is achieved. **(C)** An example of four switching targets. In a switching experiment the rat is required to switch between eight such pseudorandom targets.

The joystick hardware comprised of a commercial miniature self-centering joystick component (STD-2603AR, Element14), with two potentiometers governing movement in the X and Y axes. The joystick component was encased in a custom-made outer shell constructed from molded plastic and stainless steel, to provide protection and increased accessibility for the rats (Figure 1A). The joysticks were attached to grid floors in operant chambers (Med Associates Inc.) and interacted with the Med-PC system via a custom-made interface. Position signals from the joysticks were read from the microcontroller board (Arduino Mega 2560) by a computer running custom software developed in Matlab by the authors. This software controlled the configuration of each joystick's space and allowed the experimenter to set target areas (Figure 1B). Target areas that were positioned outside of the perpendicular 0–180° and 270–90° lines were made slightly larger, as these areas required a small amount of additional force to manipulate the joystick toward, due to the mechanical construction of the joysticks.

Several online processing functions were performed while tracking the joystick's position. For instance, during certain stages it was possible for an invalid hit to occur if a rat released the joystick at full extent and the momentum of the self-centering mechanism caused the joystick to “overshoot” the center position and enter a target at the opposing angle. To compensate for this, detection of position changes (i.e., velocity) exceeding those found to be physically possible for a rat, caused the program to enter a “silent” mode for 500 ms during which hits could not occur.

### Experimental phases

Rats completed two pre-training phases before beginning the joystick task. In the first pre-training phase food-reinforced rats completed a 1-h session where a variable interval (inter-trial interval of 10 to 120 s) scheduled a light (operant chamber center wall light) and food pellet release in the hopper. The light remained illuminated until a nose-poke event indicated that the pellet had been recovered from the food hopper. Rats receiving BSR were instead trained with a basic lever-pressing task during this phase. A lever press delivered current that increased in 10–20 μA steps every min in order to determine the value that just maximized their pressing rate—the “reward current” (Reynolds et al., 2001). A light above the lever was illuminated following a lever press to form an association between a visual sensory stimulus and the reward delivery. One-hour sessions were conducted daily until the reward current for each rat remained stable within 10% for three consecutive sessions. The reward current determined in this way for each rat was used for all subsequent experiments.

In the second pre-training phase, both food and BSR rats completed sessions in which the light was presented at a variable interval (with an inter-trial interval of 10 to 120 s) to indicate the availability of reward at the food hopper—either a food pellet or BSR. The light remained illuminated until a nose poke event, which triggered reward delivery. This task was used as an intermediate step to condition the rat to the appearance of the light indicating the availability of reward via nose poke.

In the next stage of training, rats learned to manipulate the joystick to trigger a light onset that indicated reward availability at the food hopper. During the first session, experimenter-delivered reinforcement (a light, and reward availability at the food hopper) was triggered when the rat was seen (via an infrared camera) to touch the joystick. This continued until the rat made five independent joystick movements. During 1-h sessions, run 5 days per week, training progressed through stages of increasing difficulty to achieve milestones 1 to 4, and then onto the switching phase.

Milestones were formed from standardized groups of target segments (Figure 1B). With software monitoring the joystick's position, the rats achieved what was defined as a “hit” when they moved the joystick into a target region, during any phase of joystick movement. A miss was recorded when a joystick movement departed from a small central “home zone” and then returned to this home zone without first encountering the target region. Following a hit, no other hits or misses could be recorded until the joystick returned to the home zone. There was no limit to the time taken for a rat to complete a single trial, but in practice the rats typically released the joystick within 10 s of unsuccessful exploration, and also immediately upon obtaining a hit. To pass milestones, rats were required to meet the following criteria. For milestone 1 the rats had to achieve greater than 80 hits. To pass milestones 2, 3, and 4 rats were required to achieve more than 100 hits, with a percentage of hits greater than 30% for at least 15 contiguous min. These criteria were established through pilot studies and indicated a high level of learning.

In the switching phase rats completed five sessions. Within each session the rats progressed through a unique set of 8 targets selected pseudorandomly (Figure 1C), with each rat completing the same progression of target sets. Within a session, rats were automatically moved to the next target in the set if they achieved two hits per min for three consecutive min. Session times were 1 h, or finished as soon as the rat completed all 8 targets in a set.

At the completion of experimental testing, rats received an overdose of sodium pentobarbital (1 ml/kg i.p.) and the brains of rats that received BSR were extracted. Electrode positions were mapped after histological analysis and are shown in Supplementary Figure 1.

### Data analyses

In several analyses individual performance values for rats that took different numbers of sessions to complete milestones were normalized to create mean performance values for the groups. An individual rat's entire sequence of movements within a milestone, across sessions, was divided into five equally-spaced periods. Five periods were selected as this corresponded with the mean number of sessions required to pass a milestone.

In the switching phase analysis, the time required to move the joystick into the target region was measured for each hit in each block completed (of 8 possible). The search time was defined as the time between the first joystick movement after a reinforcement and the next reinforcement. Search times within each block were then divided into five bins, and a mean search time value was calculated for each of the five time points within a block.

Custom Matlab programs were used for all behavioral data analysis. All statistical analyses were performed in Prism 6 (GraphPad Software) or SPSS Statistics 22 (IBM).

## Results

Of the 18 rats that entered the joystick task, three food and two BSR rats failed to learn the task and were excluded from analyses. Task learning was defined as meeting the criteria for achieving milestone 4 within 6 sessions. Notably, the two rats working for BSR that were excluded had the most anterior dorsal electrode placements (Supplementary Figure 1).

### Task learning

Rats engaged with the joystick using a variety of strategies. Most frequently, the forelimbs were used, with one paw performing the majority of the push or pull movement while the other paw was used for support (Figure 1A), or both paws were used together to manipulate the joystick. Overall, pushing actions were more common than pulling ones. Although individual rats appeared to have preferred approach and movement strategies, this would often change within and between sessions depending on the target position and how the joystick was approached. A few rats (one food-reinforced and one BSR-reinforced) adopted a “full body” strategy, in which they would move the joystick in a particular direction by climbing over it. Later when the target positions required more accuracy, both rats developed more refined forelimb-based strategies to manipulate the joystick.

**Figure 2:**
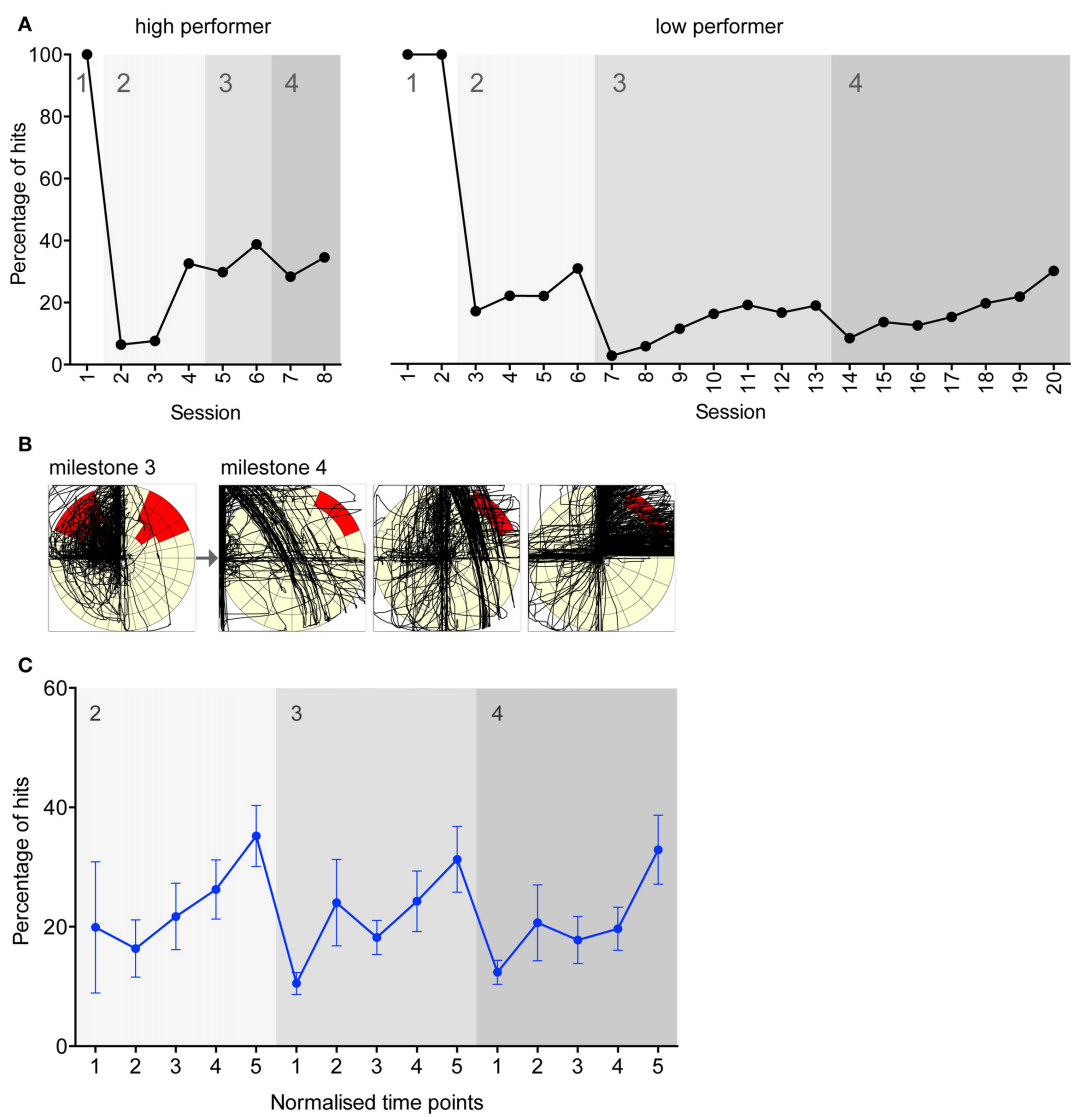
**Evidence of learning in experiments directed toward achieving each milestone. (A)** Representative accuracy performance plots for a high and low performing rat working for food reinforcement. Experiment number is shown on the x-axis, and the y-axis indicates the number of hits as a percentage of total joystick movements. Grayscale blocks indicate the milestone transitions. 100% of hits were achieved in milestone 1 as any movement resulted in a hit in this milestone. **(B)** A representative example of a single rat learning a new target position. The leftmost plot is from the final session under milestone 3, and the three consecutive plots are from the first three sessions under milestone 4. The black lines trace the joystick movements performed by the rat. **(C)** Mean percentage accuracy for all food rats. On the x-axis, five normalized time points within each milestone are shown. Error bars indicate S.E.M.

Evidence of task learning was displayed through the cycle of accuracy measures typically recorded as rats progressed (Figure 2A). Marked individual differences in learning to manipulate the joystick were found between rats, although typically accuracy increased throughout a milestone and decreased abruptly when the rat progressed to the next milestone. The learning evident in progression between milestones can also be visualized via a typical example of activity plots of a rat's joystick movements (Figure 2B). After a change from a well-learnt milestone to a new milestone, the joystick movements became more exploratory and varied, until the new milestone was converged upon and the movements became more uniform toward the target. From a behavioral perspective, a shaping process was occurring in the animal, where complex sequences of movements are refined down to the specific movements sufficient to cause the event (Ferster and Skinner, 1957).

To illustrate mean task learning across rats, normalized time points within each milestone were created to group rats with different numbers of sessions completed in each milestone (Figure 2C). The learning progression pattern seen in the individual example is also evident across all rats. For all milestones the slope is significantly non-zero (linear regression; *p* < 0.05), indicating significantly improving performance over time within milestones.

### Brain stimulation reward

**Figure 3:**
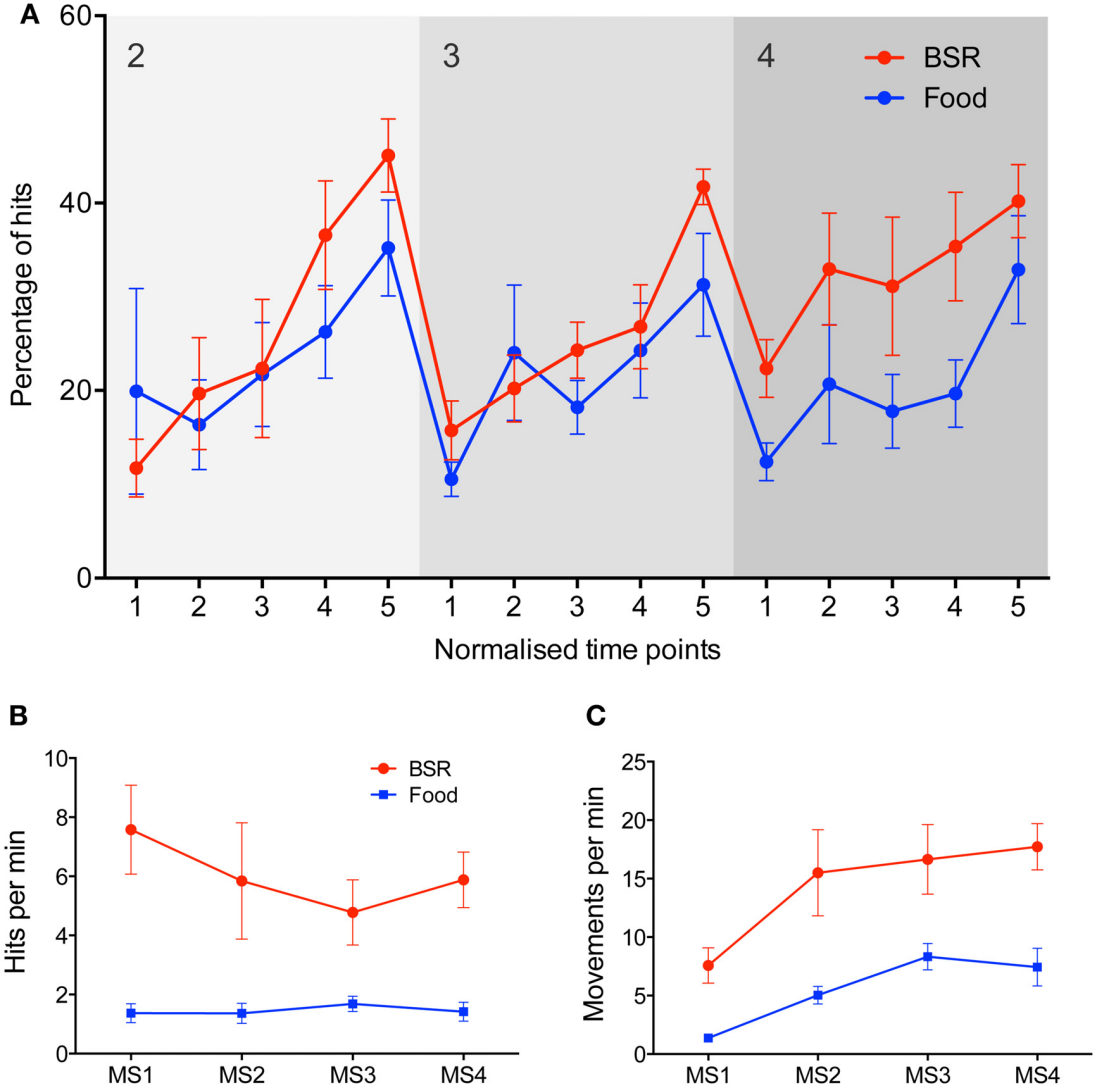
**Comparisons between food and BSR reinforcers in joystick task performance. (A)** Group average performance across milestones 2–4, with five normalized time points within each milestone on the x-axis. The y-axis represents accuracy–the percentage of hits out of all joystick movements. No significant difference in accuracy was found between food (blue, *n* = 6) and BSR (red, *n* = 7) rats [Three-Way repeated measures ANOVA, *F*_(1, 11)_ = 4.09, *p* > 0.05], although there was a trend to higher accuracy by BSR-reinforced rats (*p* = 0.068), which is particularly evident in milestone 4. A significant main effect of normalized time point was found [*F*_(4, 44)_ = 26.12, *p* < 0.05], indicating learning progression through milestones. **(B)** Mean hit rate, defined as hits per min, across each milestone in the joystick task for food (blue, *n* = 6) and BSR (red, *n* = 7) rats. BSR produced a significantly higher hit rate than food reward [*F*_(1, 44)_ = 33.05, *p* < 0.05]. **(C)** Mean movement rate, defined as joystick movements (hits or misses) per min, across each milestone in the joystick task for food (blue) and BSR (red) rats. BSR rats performed significantly more joystick movements than food reward rats [*F*_(1, 44)_ = 32.99, *p* < 0.05]. Error bars indicate S.E.M.

To investigate the versatility of the task, and its sensitivity to reinforcement type, a second group of rats received BSR in place of food as the reinforcer. Rats working for BSR progressed through the task in a similar fashion to the rats reinforced with food (Figure 3A). No significant difference in performance due to reinforcement type was found [Three-Way repeated measures ANOVA, *F*_(1, 11)_ = 4.09, *p* > 0.05], although there was a trend to higher accuracy by BSR-reinforced rats (*p* = 0.068), which is particularly evident in milestone 4. A significant main effect of normalized time point was found [*F*_(4, 44)_ = 26.12, *p* < 0.05], indicating learning progression. The two groups also took approximately the same time to complete the full task (*t* test, *M*_food_ = 17 ± 2.1 hours, *M*_BSR_ = 16 ± 1.7 hours, *p* > 0.05).

To further compare the performance of food and BSR rats, the hit rate (hits per min) was analyzed in experiments collapsed across milestones (Figure 3B). BSR produced a significantly higher hit rate than food reward [Two-Way ANOVA, *F*_(1, 44)_ = 33.05, *p* < 0.05], and this effect did not vary significantly by milestone [*F*_(3, 44)_ = 0.44, *p* > 0.05]. To determine if this difference was mediated by a general increase in activity, the rate of any joystick movement for each milestone was analyzed (Figure 3C). BSR rats performed significantly more joystick movements than those with food reward [*F*_(1, 44)_ = 32.99, *p* < 0.05]. Hence there is evidence that the increased hit rate seen in BSR rats could be due to a general increase in joystick movement rate.

We found no significant relationship between the maximum lever press rate in the pre-training continuous reinforcement lever-pressing task and later performance in the joystick-switching phase (*R*^2^ = 0.24; *p* > 0.05). The lack of relationship here indicates that the reinforcement value, for which lever-pressing rate is partially a proxy (Hodos and Valenstein, 1962), is not likely a determining factor of performance in the joystick task. Additionally, the lack of correlation suggests that the electrode placement was not a significant factor in joystick task performance.

### Behavioral switching

**Figure 4:**
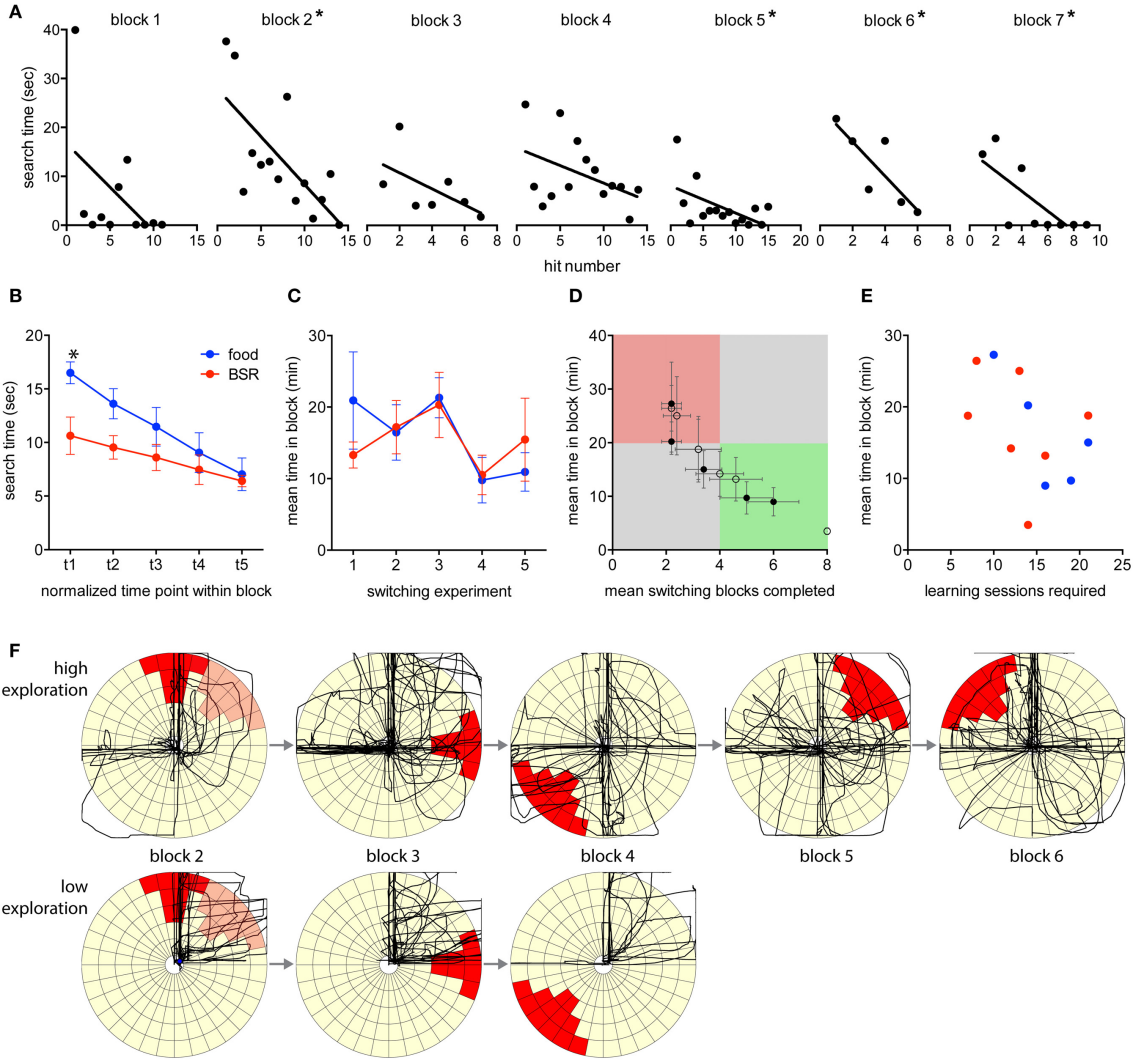
**Switching performance by blocks. (A)** Learning within the switching blocks is demonstrated by an example progression from an individual rat. The search time required to find the target was measured for each hit in a progression of blocks. Asterisks indicate blocks with a significantly non-zero slope (linear regression, *p* < 0.05). **(B)** Search times were normalized into five time points within a switching block, and then mean values for all blocks, of food- and BSR-reinforced rats were created. Search times significantly decreased in both food- and BSR-reinforced rats [Two-Way RM ANOVA, *F*_(4, 40)_ = 19.9, *p* < 0.05], indicating that target positions were being learnt. There was a significant interaction of time and reinforcement type [*F*_(4, 40)_ = 2.9, *p* < 0.05], and multiple comparisons testing revealed a significant difference between food- and BSR-reinforced rats at the start of blocks on average (Bonferroni method, *p* < 0.05). **(C)** No significant differences were found between mean performance, by time in block, of food- and BSR-reinforced rats across switching experiments [Two-Way ANOVA, *F*_(1, 42)_ = 0.04, *p* > 0.05]. **(D)** Overall individual switching performance values for food (closed circles) and BSR (open circles) rats are plotted. The green quadrant represents high performance, the red quadrant poor performance, and the gray areas represent average performance. Notably, the rat represented by the far bottom-right open circle completed all 8 blocks in the shortest possible time (3 min) in every session, and hence has no error in the mean values. **(E)** No significant correlation was found between the number of sessions required to learn the joystick task and later switching performance, as measured by mean time in block (*R* = −0.46, *p* = 0.13). **(F)** Comparing discovery-related activity plots between high and low performing rats. The first two min of activity of each block is plotted for a high performing rat that exhibited a high number of exploratory movements, and a low performing rat that exhibited limited exploratory movements during the same block progression. For reference, the previous target of block 1 is indicated in light red on the block 2 plot. Error bars indicate S.E.M.

The time required to find each target (the search time) decreased during individual switching blocks, indicating that learning was occurring within blocks (Figure 4A). The mean within-block search times for both food- and BSR-reinforced rats significantly decreased [Two-Way repeated measures ANOVA, *F*_(4, 40)_ = 19.9, *p* < 0.05; Figure 4B]. There was no significant main effect of reinforcement type [*F*_(1, 10)_ = 3.3, *p* > 0.05] although there was a significant interaction of time and reinforcement type [*F*_(4, 40)_ = 2.9, *p* < 0.05]. Multiple comparisons testing revealed a significant difference between food- and BSR-reinforced rats at the start of blocks on average (Bonferroni method, *p* < 0.05; Figure 4B).

Overall performance in this phase was measured in terms of the mean time spent in each completed block. Higher performing rats completed blocks in less time. No significant differences in performance were found between food- and BSR-reinforced rats across switching experiments [Two-Way ANOVA, *F*_(1, 42)_ = 0.04, *p* > 0.05; Figure 4C]. Additionally, there were no significant differences in performance between the five switching sessions [*F*_(4, 42)_ = 1.97, *p* > 0.05]. This indicates that the rats were not improving in their ability to dynamically switch behavior, at least within five sessions, and supports the idea of continuous new learning.

Performance can also be measured by the number of blocks completed in each experiment. Higher performing rats completed more blocks within the 1-h session. Combining these two measures allowed individual rats to be more accurately categorized by performance (Figure 4D). As an indication of the sensitivity of the task, there was a wide spread in performance, with the majority of rats performing in the middle, several high performers and one low performer. This distribution indicates that the task design was appropriately sensitive to capture a large range of individual learning abilities. To investigate individual learning in more detail we determined if the rate of learning the joystick task was an indicator of efficient switching later (Figure 4E). However, the number of learning sessions required and the mean number of switching blocks completed were not reliably correlated (*R* = −0.46, *p* = 0.13). Although, there was a trend to larger numbers of learning sessions being associated with less time to complete switching blocks. Also notable was the finding that BSR and food reinforcement, in both the joystick learning and switching phases, were comparable.

Regardless of reinforcer, it was commonly observed in the switching experiments that behavior was seemingly packaged into sets. It was common for rats to have learned a number of techniques to achieve hits in particular target locations. These techniques would involve approaching the joystick from a particular angle, in a particular manner; using a distinct paw, or head, or body, configuration to push or pull the joystick in a stereotypical manner; and finally withdrawing from the joystick to collect the reward with a further stereotyped movement. It was also common to observe rats switching between several previously learnt behavioral routines once the target changed. If no existing routine achieved the goal, it was common for high performing rats to enter a presumed “exploration mode” involving trial and error attempts, which may result in learning a novel action sequence. Low performing rats would tend to perseverate with previously learnt target locations, or give up trying. Plotting the first two min of activity for each block performed by two rats, a high performer and a low performer, illustrates this distinction (Figure 4F). During this crucial early time period the high performing rat exhibited a larger number of exploratory movements and rapidly found new targets, while the low performing rat exhibited limited exploratory movements and eventually failed to find a novel target, failing the task sooner.

## Discussion

### Task performance

Performance measures indicated that the majority of rats were able to learn the joystick task, which involved motor coordination and spatial cognition far beyond that required for comparatively simpler tasks such as lever pressing. Rats were able to progress through increasingly difficult task demands, eventually learning the action required to move the joystick to a precise spatial location that triggered reinforcement. Moreover, with individual variability, they could discover new actions to locate new target areas. The observed shaping of responses to new targets, from widely distributed, complex movements to more precise, elegant movements are similar to those found in humans on a similar action discovery task (Bednark et al., 2013; Bednark and Franz, 2014). This demonstrates the versatility of the present task.

The stringent cognitive and physical demands imposed by this task were exemplified in the three food rats and two BSR rats that failed to meet the early stage learning criteria required to continue. The high task demands were also evident in what seemed to be low accuracy rates for rats that completed the task. However, it is important to appreciate that “misses” with the joystick are easy to accrue for rats. For instance, a rapid nudge of the joystick may move it out of the home zone, back through the home zone, and then briefly out again to accrue two misses in quick succession. Especially in the case of rats with less refined control of the joystick, the hit percentage scores became negatively skewed. Finally, the targets in milestone 3 and 4 represented approximately 22% and 6%, respectively of the total movement area. Consequently, our use of small targets necessitated precise and repeatable movements. This made the joystick task challenging for rats, who were unlikely to have prior experience of generating such movements. The inherent difficulty of the task did however allow for a wide range of performance values, and hence was able to discriminate the different action discovery and selection abilities of individual subjects.

### Food vs. BSR

In most respects, reinforcement with food or BSR did not differentially affect task performance. Both BSR- and food-reinforced rats showed similar learning profiles throughout the task, and took similar average times to complete the overall sequence of tasks. BSR-reinforced rats exhibited a significantly higher hit rate than food rats, which was likely due to a significant increase in joystick movements in general. Additionally, rats receiving food pellets had to take the time to consume the pellet before returning to the joystick, which would also contribute to the joystick movement rate difference. It was surprising that the greater movement rate seen in BSR rats did not translate into higher accuracy performance, although it may have contributed to the non-significant trend to higher performance of BSR rats apparent in milestone 4. In a task such as this, an increase in random movements might signify increased levels of exploration, which in a human version of this task (Stafford et al., 2012) leads to higher endpoint levels of performance. That this was not statistically reliable in the present study suggests that the additional movements of BSR rats may not reflect task-related exploration, rather it could have represented a general state of increased vigor.

Our results are therefore consistent with earlier findings (Panksepp and Trowill, 1967; Trowill et al., 1969) indicating that BSR has comparable learning characteristics to natural rewards when experimental contingencies are made consistent, and is not somehow promoting unnatural learning abilities. The partial differences produced by BSR could easily result from small differences in reinforcement value, and perhaps sweeter food or relatively lower stimulating current could have abolished the differences.

A further consideration on the similarity of performance between food- and BSR-reinforced rats is that the secondary reinforcer (the light) was the same in both conditions. As a well-learnt secondary reinforcer is able to exert considerable control over behavior (Hull, 1943; Davis and Smith, 1976), the overall reinforcement value provided to the rats may have been similar. Differences between reinforcement groups could be due to BSR being more effective at sustaining the secondary reinforcing properties of the light.

### Learning theories

The joystick task presented here encompasses the concepts embodied in multiple theories of learning. This is similar to real-world tasks, where it is difficult if not impossible to precisely discern the contributions of each type of learning to a task. In the present task, for instance, there is classical conditioning of the light stimulus by the reward delivery. Additionally, rat behavior to activate the light and receive the reward is likely governed, at least in part, by goal-directed learning. To define the task as goal-directed would require it to meet several criteria (Heyes and Dickinson, 1990; de Wit and Dickinson, 2009). Most importantly, the task must be instrumental–it must be controlled by the causal relationship between the action and its outcome, as opposed to predictive relationships between stimuli and the outcome. The joystick task satisfies this criterion as the target area of the joystick is constantly changing, especially in the switching phase, and no stimuli predict the correct action to perform.

However, it is difficult to distinguish between a system of continually updating stimulus-response associations, defined and redefined by reward delivery, and pure theoretical goal-directed learning. For instance, in the joystick task it could be argued that when a particular action becomes well learnt within a session, then more habitual, stimulus-response processes would take over. A further criterion of goal-directed learning that can help in elucidating this is that the actions of the animal in attaining the goal should be modulated by its motivational appraisal of the outcome. This is typically shown experimentally by devaluing a specific, previously learned reward prior to a test session, and then measuring an immediate reduction in responses to receive that particular reward, but not other rewards (de Wit and Dickinson, 2009). Although potentially difficult, this could be tested in the BSR joystick task by using two instrumental actions relating to two target areas with different rewards, and devaluing BSR before the test session via association with an aversive affective state, such as nausea. Although we did not perform this test, it has been shown previously that a multiple lever pressing task can meet this criterion for goal-directed learning (Dickinson et al., 1996), and as such it can be inferred that at least part of the joystick task would do likewise.

### Behavioral switching

In the switching phase, rats were able to adjust to changing task demands and discover new actions required to achieve the reward. The hypothesis that the switching phase assays action discovery processes was supported by evidence that high performing rats demonstrate a higher degree of exploratory movements to new spatial locations at the start of a block—an apparent “discovery mode.” If the exploratory movements were reinforced it could result in learning a novel action sequence to be repeated later. That the switching phase involves action discovery was also supported by the lack of improvement in performance seen over the five switching experiments. This indicates that rats are required to learn new actions to achieve the reward.

The broad distribution of individual performance revealed in the switching phase demonstrates the utility of the joystick task in discriminating between natural learning abilities of rats. Because the factors of reward type and electrode placement have been ruled out, it is more likely that individual joystick task performance is due to a combination of different intrinsic motivations, intelligence for this type of task, and motor coordination. Moreover, importantly for studying action discovery and selection, this broad performance distribution is clearly demonstrated in the switching experiments, where it is thought that action discovery and selection are most closely modeled due to the constant learning required.

The individual differences seen in switching performance seemed to be independent of the individual differences in initial learning of the joystick task. However, there was a trend suggesting that a greater number of learning sessions performed was associated with less time in switching blocks. This could indicate that greater experience with the joystick might improve later switching performance. Alternatively, the lack of any reliable correlation between initial learning and subsequent switching could imply that these two competences are subserved by different cognitive abilities. This division could relate to habitual vs. goal-directed systems controlling behavior, or the ability to transition between them (circuits reviewed in Redgrave et al., 2010; Burguière et al., 2014; Gillan and Robbins, 2014). This analysis suggests that an extended learning phase would establish habit-driven stimulus-response control for effective performance with a relatively static target location. On the other hand, the switching phase of the task would necessitate a change to a goal-directed approach. The processes of habit development and switching back to goal-directed control may be partially independent and therefore differentially subject to individual variation.

It is also possible to characterize habitual vs. goal-directed control within the switching phase. After the target area has been shifted, rats prepared to explore the joystick movement space appear to be operating under goal-directed control, while those reluctant to shift from a previously reinforced target area seem to be behaving habitually, despite the now devalued outcome. The goal-directed, habit-driven dichotomy, and the transition between them, has been extensively studied in animal models, often in the context of substance dependence (e.g., Everitt and Robbins, 2005) and disease states such as obsessive-compulsive disorder (e.g., Gillan et al., 2011). While previous studies have used simple tasks such as lever pressing the current joystick task offers a richer behavioral domain within which the development of habits and the transitioning back and forth between goal-directed and habitual control could be analyzed with repeated measures designs. When combined with recently developed technologies for cell-specific excitation and silencing (Tye and Deisseroth, 2012), the task could be used to more precisely elucidate the neural circuitry responsible for these important processes.

The switching phase of the joystick task could also be used to complement previous analyses of cognitive and behavioral flexibility, which have used task/set switching tasks, or discrimination and reversal learning paradigms (e.g., Floresco et al., 2006; Block et al., 2007; Coppens et al., 2010; Richter et al., 2014). In the joystick task, if the actions associated with specific targets in a switching experiment are viewed as tasks to perform with the joystick, once a target is switched to the next position the rat must engage in a form of cognitive task switching. It must flexibly disengage from the present task of performing actions associated with receiving a reward in the previous target location, and switch to or discover a new task associated with the new target. This would be uncued task switching as the rat is given no indication of a switch in target location other than the success or failure of their movement at triggering the light stimulus. However, switching in the joystick task could easily be cued by another stimulus. Impairments in behavioral flexibility are commonly found in Parkinson's disease (Shook et al., 2005), obsessive-compulsive disorder (Gu et al., 2008), and schizophrenia (Floresco et al., 2009). The present joystick task could serve as a tool to aid investigations of the cognitive processes affected by these conditions, and of the underlying mechanisms in appropriate animal models.

### Summary

Results presented here suggest that this joystick task can be used to model action discovery and selection in the rat. The switching phase is the most relevant aspect of the task, as rats were forced into goal-directed mode to discover the action required to elicit the light and reward. Moreover, it was common during the switching phase for rats to learn behavioral routines that they selected between once the target was changed. If no existing routine achieved the goal, a novel action sequence was discovered through trial and error.

Behavior in natural environments invariably consists of changing conditions, to which the appropriate response must be learnt through feedback and behavioral adjustment. A higher degree of environmental feedback and interaction in a task makes it more analogous to natural behavior outside the lab (Cisek and Kalaska, 2010). With progression through the joystick task, changing target locations demand that multiple actions be learnt from which the animal must then select an action that will achieve the reward. This relatively high degree of environmental interaction provides the joystick task with ecological validity, and is central in its utility for investigating action discovery, selection, and the behavioral flexibility impairments seen in conditions such as Parkinson's disease and obsessive-compulsive disorder.

### Conflict of interest statement

The authors declare that the research was conducted in the absence of any commercial or financial relationships that could be construed as a potential conflict of interest.

## Supplementary material

Supplementary Figure 1 **Electrode positions from histology of BSR rats**. Illustration of a sagittal view of the substantia nigra *pars compacta* (SNc) and surrounding area, based on features found at 1.8 mm lateral to Bregma. The colored markers represent the approximate center positions of electrode tips. The color scale indicates the maximum number of lever presses performed during pre-joystick training. Markers with asterisks indicate rats that did not meet criteria to complete the joystick task. SNr, substantia nigra *pars reticulata*; cp, cerebral peduncle; PSTh, parasubthalamic nucleus; ml, medial lemniscus.
