# Supplementary figures and images for: A behavioral task for investigating action discovery, selection and switching: comparison between types of reinforcer
Source: Front Behav Neurosci. 2014 Nov 18;8:398. doi: 10.3389/fnbeh.2014.00398 (PMC4235381; doi:10.3389/fnbeh.2014.00398)

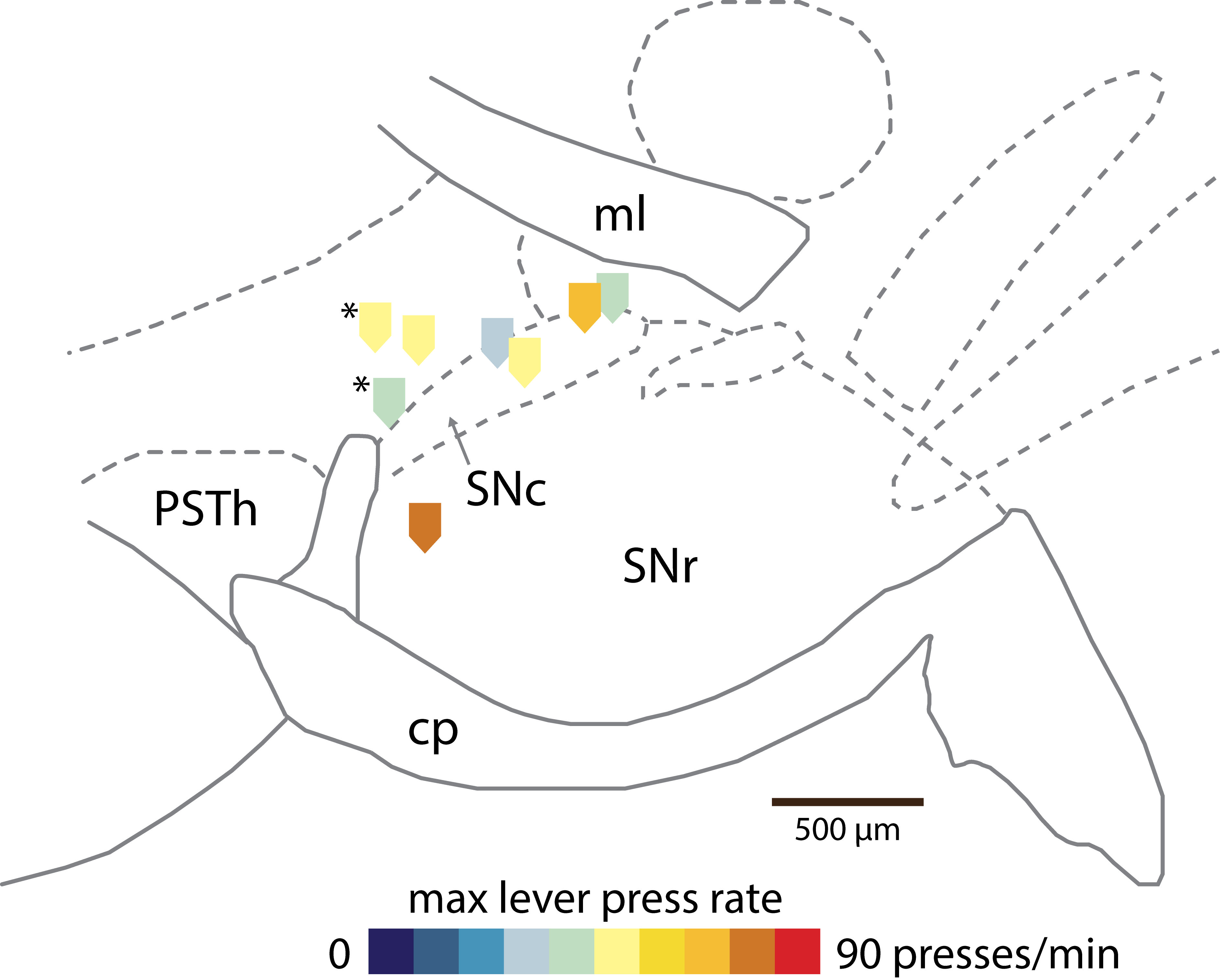

Supplement: Supplementary Figure 1 — Electrode positions from histology of BSR rats. Illustration of a sagittal view of the substantia nigra pars compacta (SNc) and surrounding area, based on features found at 1.8 mm lateral to Bregma. The colored markers represent the approximate center positions of electrode tips. The color scale indicates the maximum number of lever presses performed during pre-joystick training. Markers with asterisks indicate rats that did not meet criteria to complete the joystick task. SNr, substantia nigra pars reticulata; cp, cerebral peduncle; PSTh, parasubthalamic nucleus; ml, medial lemniscus. [file Image1.TIF]
